# Examining negative urgency as a predictor of eating disorder maintenance in purging syndromes

**DOI:** 10.1002/jclp.23683

**Published:** 2024-03-30

**Authors:** Sarah A. Horvath, Pamela K. Keel, K. Jean Forney

**Affiliations:** 1 Department of Psychology, Ohio University, Athens, Ohio, USA; 2 Department of Psychiatry, University of Colorado School of Medicine, Aurora, Colorado, USA; 3 Department of Psychology, Florida State University, Tallahassee, Florida, USA

**Keywords:** bulimia nervosa, eating disorder maintenance, negative urgency, personality, purging disorder

## Abstract

**Objectives:**

Negative urgency is associated with short-term maintenance of binge eating and purging in unselected samples. The current study used an eating disorder sample to test the hypothesis that negative urgency maintains bulimia nervosa (BN) and purging disorder (PD) at long-term follow-up. It was also hypothesized that baseline differences in negative urgency between BN and PD would remain at follow-up.

**Methods:**

Secondary analyses were conducted on a sample of women who engaged in recurrent self-induced vomiting (*n* = 68; 52.9% BN; 47.1% PD). Women completed diagnostic interviews and questionnaires at baseline and at a mean (SD) of 5.95 (1.58) years follow-up (range = 2.51–9.62; retention rate = 75%).

**Results:**

Negative urgency did not predict eating disorder diagnostic status, recovery status, or global eating pathology at follow-up (*p*’s = .06–.83). There were no significant differences in negative urgency across women with BN and PD at follow-up (*p* = .16). However, post hoc analyses indicated that negative urgency was not stable across time (ICC = .102). Increases in negative urgency from baseline to follow-up were associated with greater global eating pathology at follow-up (*p* = .002).

**Conclusion:**

Results suggest negative urgency does not predict long-term eating disorder maintenance. Negative urgency may not be a stable personality trait but rather an indicator of overall poor emotion regulation. Future research should confirm that changes in negative urgency predict chronic eating pathology over long durations of follow-up in women who have *increasing* negative urgency across time.

## 1 | INTRODUCTION

Negative urgency, or the tendency to act impulsively in response to distressing emotions, is a personality factor associated with binge eating and purging behaviors (Cyders & Smith, 2008). This transdiagnostic personality factor is a posited risk and maintenance factor for binge eating and purging in unselected samples (Davis et al., 2024; Fischer et al., 2008; Pearson et al., 2012; Wenzel et al., 2014). We are unaware of data testing negative urgency as a long-term maintenance factor for eating disorder syndromes characterized by these behaviors—bulimia nervosa (BN; characterized by both recurring binge eating and compensatory behaviors and the absence of low body weight) and purging disorder (PD; characterized by recurring purging behaviors and the absence of low body weight). BN and PD are distinguished by the presence of DSM-5 (American Psychiatric Association, 2013) binge-eating episodes (objectively large, out of control eating episodes; e.g., a large pizza) in BN and the absence of DSM-5 binge-eating episodes in PD. Many individuals with PD experience subjective binge-eating episodes, or loss of control while eating a small or normal amount of food (e.g., two slices of pizza; Forney et al., 2014). Elevated negative urgency in BN compared to PD (Davis et al., 2020) contribute to data supporting the concurrent validity of these diagnoses. If these group differences are maintained across time, this would support negative urgency as a stable personality factor that consistently differentiates BN and PD. More broadly, if negative urgency maintains eating disorders longitudinally, measures of negative urgency could identify individuals at high-risk for a chronic course. The current study examines the longitudinal association between negative urgency and eating disorder outcomes and investigates the stability of diagnostic differences in negative urgency at an average of 6-year follow-up. We also investigate whether negative urgency demonstrates stability over long-term follow-up—a premise that has not been directly tested to our knowledge.

Theory proposes that binge eating and purging behaviors represent urgent attempts to mitigate negative affect despite their potential harmful consequences (Pearson et al., 2015; Polivy & Herman, 1993). Broadly, data suggest that negative urgency is a short-term risk factor for developing binge eating and purging behaviors (Davis et al., 2024; Pearson & Smith, 2015; Puccio et al., 2019) and changes in negative urgency maintain these behaviors in the short-term (i.e., 3–4 weeks; Anestis et al., 2007). Specifically, negative urgency has been replicated as a short-term risk factor for developing binge eating and purging over time periods ranging from 1 month to 1 year in samples including early adolescent girls (i.e., 5th–6th grade; Pearson & Smith, 2015; Pearson et al., 2015), undergraduate men and women (Anestis et al., 2007; Davis et al., 2024; Wenzel et al., 2014), and women from the community (Puccio et al., 2019). Understanding how negative urgency is related to long-term maintenance may help identify who is at risk for a chronic illness course. If negative urgency is associated with increased risk for maintaining eating pathology long-term, results would highlight the importance of targeting underlying emotion regulation and distress tolerance deficits within eating disorder treatment.

Negative urgency and impulsivity are correlated with the frequency of objective and subjective binge eating within BN and PD (Brown et al., 2011; Forney et al., 2014, 2016) and women with BN and PD tend to endorse more impulsivity than women without eating disorders (Brown et al., 2011; Davis et al., 2020; Krug et al., 2020, 2022). Further, negative urgency is elevated in women with BN relative to PD (Davis et al., 2020), potentially reflecting that individuals with BN engage in more frequent eating disorder behaviors compared to individuals with PD (Forney et al., 2014, 2016). This finding may be unique to negative urgency relative to other aspects of personality (Brown et al., 2011; Davis et al., 2020; Krug et al., 2022). Given that personality characteristics are perceived as relatively stable (Cobb-Clark & Schurer, 2012), these findings suggest that negative urgency may be a specific personality characteristic relevant for distinguishing between BN and PD.

Negative urgency comprises the combination of urgency *and* negative affect, thus making it more difficult to determine which component primarily impacts the maintenance of eating pathology and the stability of negative urgency across time. The association between negative urgency and binge eating and purging remains significant after adjusting for negative affect in cross-sectional research (Racine et al., 2013; Wenzel et al., 2014). Given that negative urgency involves acting rashly when experiencing negative affect, adjusting for negative affect, an established risk and maintenance factor for eating pathology (Stice, 2002), provides a stronger test of the urgency component inherent in negative urgency. Risk and maintenance models of negative urgency do not adjust for negative affect, which limits our understanding of the mechanism driving the association between negative urgency and binge eating and purging.

## 2 | THE CURRENT STUDY

The current study explored whether negative urgency is a maintenance factor for eating disorders characterized by purging. Given the positive relationship between negative urgency and eating pathology (Cyders & Smith, 2008; Davis et al., 2020; Forney et al., 2014, 2016), we hypothesized that greater baseline negative urgency in individuals diagnosed with BN or PD would be associated with greater likelihood of an eating disorder diagnosis at 6-year follow-up, a reduced likelihood of recovery, and greater global eating pathology at 6-year follow-up. In the parent studies (Forney et al., 2021, 2022), three approaches were taken to operationalize outcome to account for limitations of each operationalization. Eating disorder diagnosis was chosen to characterize illness status. Second, an empirically-based approach to recovery (Bardone-Cone et al., 2010) was utilized, as the absence of illness does not imply recovery. Finally, given recognition that eating pathology is a dimensional construct and dimensional approaches may have more clinical utility than categorical approaches to diagnosis (Wildes & Marcus, 2013), global eating pathology was used as a continuous outcome. Baseline data from the same sample (Davis et al., 2020) demonstrated greater negative urgency in those with BN relative to PD; therefore, we posited that individuals with BN at baseline would continue to endorse greater negative urgency at follow-up, compared to individuals with PD at baseline. Finally, we examined whether statistically significant results were supported after adjusting for negative affect, to provide a stronger test of the urgency inherent in negative urgency. Results from the present study will help determine if negative urgency functions as an effective personality factor for identifying individuals vulnerable to chronic eating disorder course and address the validity of the BN-PD distinction.

## 3 | MATERIALS AND METHODS

### 3.1 | Participants and procedure

Baseline data come from a prior study examining psychological and physiological correlates of binge eating (Davis et al., 2020; Keel, Eckel, et al., 2018, Keel, Haedt-Matt, et al., 2018; Keel et al., 2023). At baseline, women ages 18–45 were recruited from the community and university via emails and flyers in Iowa City, IA and Tallahassee, FL. Sixty-eight women who engaged in recurrent self-induced vomiting (36 BN; 32 PD) completed diagnostic interviews and a questionnaire battery at baseline (Davis et al., 2020; Keel, Eckel, et al., 2018, Keel, Haedt-Matt, et al., 2018; Keel et al., 2023) and were invited to participate in a larger follow-up study (Forney et al., 2021, 2022). The baseline parent study required a body mass index (BMI) between 18 and 26.5 kg/m^2^, recurrent self-induced vomiting (on average at least once weekly), and meeting criteria for DSM-5 BN or research criteria for PD. Our sample size is discrepant from Davis et al. (2020) due to inclusion/exclusion criteria for the follow-up study (Forney et al., 2021, 2022). While the baseline parent study (Davis et al., 2020; Keel, Eckel, et al., 2018; Keel, Haedt-Matt, et al., 2018; Keel et al., 2023) excluded those prescribed psychotropic medications, medical conditions, or treatments that affect appetite or weight, pregnancy or lactation, current major depressive disorder or current DSM-IV substance use disorder, the follow-up study (Forney et al., 2021, 2022) included individuals who completed the baseline assessment and met criteria for BN or PD regardless of meeting other eligibility criteria (e.g., someone ineligible for the baseline parent study based on a high BMI would still be eligible for the follow-up study). Further, only individuals with BN who engaged in recurring self-induced vomiting where invited for follow-up. Individuals who declined to be re-contacted at baseline were ineligible for the follow-up study. In the parent follow-up study, 94.5% of participants were successfully contacted at follow-up (Forney et al., 2022). Of those sought in this present subsample, three-quarters (*n* = 51) participated at a mean (SD) of 5.95 (1.58; range = 2.51–9.62) years after baseline assessment; the majority completed a phone interview and questionnaire assessments (*n* = 47), one woman completed a phone interview and no questionnaire assessments, and three women completed only questionnaire assessments. Participants were 20.78 (3.26) years of age at baseline and 26.59 (3.74) years of age at follow-up. See Table 1 for demographic information. The Institutional Review Board approved all study procedures.

## 4 | MEASURES

### 4.1 | Negative urgency

The Negative Urgency subscale from the UPPS Impulsive Behavior Scale (Whiteside& Lynam, 2001) was used to assess the tendency to engage in rash behaviors in the context of negative affect at baseline and follow-up. Sample items include: “When I am upsetI often act without thinking,” and “In the heat of an argument, I will often say things thatI later regret.” The UPPS has demonstrated convergent validity with other measures of personality and impulsivity, as well as good to excellent internal consistency in undergraduate men and women (Whiteside & Lynam, 2001). The Negative Urgency subscale has also exhibited strong test–retest reliability across two assessments over an average duration of 8.6 days in a sample of community men and women (Weafer et al., 2013). The internal consistency of the Negative Urgency subscale in the current sample was good at baseline (*α* = .85) and follow-up (*α* = .91).

### 4.2 | Eating pathology

The Eating Disorder Examination (EDE; Cooper & Fairburn, 1987) was administered at baseline and follow-up to determine the presence of an eating disorder, assess recovery status at follow-up, and measure global baseline and follow-up eating pathology, consistent with prior work using this sample (Forney et al., 2021, 2022). DSM-5 eating disorder diagnoses (e.g., anorexia nervosa, bulimia nervosa, binge-eating disorder, and other specified feeding or eating disorder) were used at follow-up. To be diagnosed with an other specified feeding or eating disorder, participants needed to report engaging in at least 12 eating disorder behaviors (e.g., objective or subjective binge-eating episodes, self-induced vomiting, laxative misuse, diuretic misuse, fasting, or excessive exercise) over the 3 months before interview. Interrater reliability was excellent, with Cohen’s *κ* ranging from 0.90 to 1.0 (Davis et al., 2020; Forney et al., 2021, 2022; Keel, Eckel, et al., et al., 2018, Keel, Haedt-Matt, et al., 2018; Keel et al., 2023) in parent studies.

Recovery status was determined using the criteria of Bardone-Cone et al. (2010). Participants were categorized as remitted if they denied binge eating, purging, and fasting in the 3 months before interview on the EDE and scored within one standard deviation of age-matched community norms on all subscales of the Eating Disorder Examination-Questionnaire (Fairburn & Beglin, 1994).

Global EDE scores were used to assess global eating pathology. Internal consistency in the current sample was good at baseline (*α* = .82) and follow-up (*α* = .88).

### 4.3 | Negative affect

The Beck Depression Inventory (BDI) (Beck, 1961) was used as a measure of negative affect at baseline. The BDI-II was used to measure negative affect at follow-up. The BDI is highly correlated with the Negative Affect subscale of the Positive and Negative Affect Schedule (PANAS; Watson et al., 1988; *r* = .58) and has demonstrated good test–retest reliability, ranging from .76 to .95 across a period ranging from 1 week to 6 months (Wang & Gorenstein, 2013). Internal consistency was good at baseline (*α* = .88) and follow-up (*α* = .93).

### 4.4 | Statistical analyses

Consistent with prior work using this sample (Forney et al., 2021, 2022) missing data for baseline variables (negative affect [*n* = 1/68]), and missing data for follow-up variables (negative urgency [*n* = 18/68], negative affect [*n* = 19/68], eating disorder diagnostic status [*n* = 22/68], recovery status [*n* = 17/68]), and global eating pathology [*n* = 25/68] were multiply imputed 40 times using the R package *mice 3.4.0* (Van Buuren et al., 2011). As a missing data approach, multiple imputation is advantageous over other missing data methods, such as expectation maximization or listwise deletion, as pooling across multiple datasets allows for the modeling of sampling error and minimizes bias due to attrition (Enders, 2010). Multiple imputation, compared to full information maximum likelihood approaches, also allows for handling missing data separately by diagnosis, which was important given hypothesized group differences (Enders, 2010). Missing data were imputed separately for women with women with baseline BN and women with baseline PD due to hypothesized group differences. Pooled binomial logistic regression models tested baseline negative urgency as a predictor of follow-up ED diagnostic status and recovery status. A pooled linear regression model tested baseline negative urgency as a predictor of follow-up global eating pathology, adjusting with baseline global eating pathology. The pattern of results was the same when adjusting for duration of follow-up; results are presented without this covariate for parsimony. A pooled *t*-test via the R package *MKmisc* (Kohl, 2022) compared negative urgency at follow-up between individuals diagnosed with BN and PD at baseline.

## 5 | RESULTS

Analyses demonstrated no significant differences in baseline negative urgency, negative affect, eating pathology, or BMI across individuals who participated in follow-up compared to individuals who did not participate, suggesting data were missing at random (all *p*’s > .24). In total, 60.1% of the sample (57.5% baseline BN; 63.4% baseline PD) met criteria for an eating disorder at follow-up and 26.2% (25.6% baseline BN; 26.9% baseline PD) met recovery criteria.

Baseline negative urgency did not predict eating disorder diagnostic status (odds ratio [OR] = 1.11, *p* = .83, 95% confidence interval [CI]: 0.41–3.03), recovery status (OR = 0.29, *p* = .06, 95% CI: 0.08–1.06), or global eating pathology (*B* = −0.35, *p* = .23, 95% CI: −0.94 to 0.23) at follow-up. Results were not significant; thus, additional analyses covarying for negative affect were not conducted.

Table 2 provides descriptive statistics for negative urgency at baseline and follow-up. There was not a significant difference in negative urgency at follow-up between individuals with baseline BN and baseline PD (*t*[41.15] = −1.44; *p* = .16; Cohen’s *d* = .35).

### 5.1 | Post hoc analyses

Negative urgency comprises the combination of negative affect and urgency; thus, a change in negative affect may have driven null findings. Pooled intraclass correlations (ICC) were used to examine the stability of negative affect and negative urgency across time, given that ICC accounts for both within subjects and group level changes across time. The stability of negative affect (ICC = .31) and negative urgency (ICC = .10) across baseline to follow-up was poor (Cicchetti, 1994).

Because negative urgency had poor reliability across time, we explored whether changes in negative urgency across baseline to follow-up were associated with outcome. Consistent with previous studies examining changes in negative urgency (Anestis et al., 2007), a simple change score was calculated and used as a predictor of outcome. Change in negative urgency did not predict eating disorder diagnostic status (OR = 1.73, *p* = .196, 95% CI: 0.74–4.04) or recovery status at follow-up (OR = 0.79, *p* = .61, 95% CI: 0.31–1.98). However, increases in negative urgency across baseline to follow-up were associated with greater global eating pathology at follow-up (*B* = 0.69, *p* = .002, 95% CI: 0.28–1.09); results remained significant after adjusting for changes in negative affect (*B* = 0.63, *p* = .007, 95% CI: 0.22–2.85).

## 6 | DISCUSSION

The current study tested negative urgency as a predictor of the long-term maintenance of eating pathology and the stability of group differences in negative urgency in women with BN and PD. Contrary to hypotheses, negative urgency at baseline did not predict eating disorder diagnostic status, recovery status, or global eating pathology longitudinally. However, post hoc analyses indicated that increases in negative urgency over time were associated with greater global eating pathology at follow-up. Negative urgency did not significantly differ across individuals with BN and individuals with PD at follow-up.

While existing literature suggests that negative urgency increases risk for, and maintains, eating pathology in the short term (Anestis et al., 2007; Davis et al., 2024; Fischer et al., 2008; Wenzel et al., 2014), results from the present study suggest that negative urgency assessed at a single time point does not predict the long-term maintenance of eating pathology. Although greater baseline negative urgency approached traditional thresholds for significance in predicting a decreased likelihood of recovery (*p* = .06), we conservatively do not interpret this as a true finding. While this finding may have reached statistical significance in a larger sample, the relative effect size achieved in the current analyses is very small (Chen et al., 2010). Our longer duration of follow-up (6 years) may explain discrepancies from prior work finding that negative urgency predicts binge eating over 3–4 weeks (Anestis et al., 2007) and 5–8 months (Davis et al., 2024). These discrepancies may also be explained by differences in unselected samples (Anestis et al., 2007; Davis et al., 2024; Fischer et al., 2008; Wenzel et al., 2014) compared to our selected sample of those with eating disorders, as impulsive personality characteristics, including negative urgency, are elevated in eating disorders (Brown et al., 2011; Davis et al., 2020; Krug et al., 2022) and therefore may be less predictive of changes in eating pathology when there is limited range.

Negative urgency was found to have low stability across baseline to follow-up in the current sample. Indeed, post hoc analyses found that increases in negative urgency longitudinally were associated with greater global eating pathology at follow-up. Thus, it may be *change*s in negative urgency, not baseline levels, that are most relevant. Individuals who experienced increases in negative urgency from baseline to follow-up likely escalated their engagement in impulsive behaviors such as vomiting or binge eating while experiencing negative affect. Recent data also suggests that fasting behaviors can contribute to increased negative urgency over time, ultimately increasing binge eating (Davis et al., 2024). Overall, the poor stability of negative urgency calls into question whether negative urgency is a stable personality trait or a malleable marker of emotion dysregulation. Future research should confirm that negative urgency is particularly relevant for predicting the maintenance of eating pathology in women who have *increasing* difficulty utilizing adaptive strategies for regulating impulsive response to negative affect. Thus, women who experience increases in negative urgency may be more likely to experience a chronic illness course.

Negative urgency at follow-up did not significantly differ across individuals with BN and individuals with PD; group differences were of a small to medium effect size. Baseline data from the same study showed that negative urgency was elevated in women with BN, relative to individuals with PD (Davis et al., 2020). At follow-up, women with PD exhibited somewhat greater—albeit not significantly greater—negative urgency relative to women with BN. Notably, the absolute effect sizes were comparable in our sample (Baseline *d* = .37; Follow-up *d* = −.35). Personality *traits* are posited to remain relatively stable across the lifetime (Cobb-Clark & Schurer, 2012); however, the current study suggests that negative urgency may instead be a fluctuating correlate of eating pathology. Indeed, post hoc analyses revealed that our indicator of negative affect (the Beck Depression Inventory) had higher stability across time than negative urgency. Results therefore suggest that targeting deficits in emotion regulation and distress tolerance may be particularly important in the treatment of individuals with binge eating and/or purging symptoms. This is consistent with data supporting Dialectical Behavior Therapy (Linehan, 1993), which targets emotion regulation, as an empirically supported treatment for BN (Rozakou-Soumalia et al., 2021). Indeed, interventions that incorporate cognitive behavioral, cognitive control, or emotion modulation/impulsivity reduction modules are effective at reducing negative urgency (Peckham et al., 2019; Weiss et al., 2015) and impulsivity in general (Peckham & Johnson, 2018) in other populations (i.e., individuals in general partial hospitalization programs and undergraduate students). As such, similar interventions may be useful for targeting negative urgency in populations with binge eating and/or purging symptoms.

The current study benefits from several strengths, including the use of a sample of women with diagnosed BN and PD, a longitudinal design spanning 6 years, high retention, measures with well-described psychometric properties, and use of multiple imputation to limit bias from attrition. This study also used numerous operationalizations of outcome including diagnostic status, recovery status, and global eating pathology. Limitations include the relatively small sample size and lack of additional data points across follow-up. Despite sample size limitations, post hoc power analyses indicated that we were powered to detect medium to large effect sizes. While medium to large effect sizes are more observable (Cohen, 2013) and may be more clinically relevant, it would be beneficial for future studies to replicate the current findings with a larger sample size powered to detect small effect sizes. In particular, the relationship between negative urgency at baseline and recovery status at follow-up approached traditional thresholds for significance, suggesting that this association should be further investigated in future studies with larger sample sizes. Additional data collection in between baseline and 6-year follow-up would have been useful for providing a comprehensive image of negative urgency and eating pathology across the years, particularly given recent data that negative urgency mediates the relationship between fasting and binge eating (Davis et al., 2024). The sample was all female, primarily white, highly educated, and in early adulthood at baseline, which limits generalizability across gender identities, racial/ethnic minority groups, education levels, and the lifespan. Future research should use a developmental approach in a more representative sample to assess negative urgency across the lifespan, sampling data at additional intervals to assess how negative urgency fluctuates across the course and severity of an eating disorder.

## 7 | CONCLUSION

Taken together, results suggest that negative urgency assessed at baseline does not predict the long-term maintenance of BN and PD. However, increases in negative urgency across baseline to follow-up were associated with greater global eating pathology at follow-up in post hoc analyses, suggesting that changes in negative urgency may be useful for predicting who maintains eating pathology long-term. While clinicians should expect negative urgency to fluctuate across time, individuals who specifically endorse an increase in impulsive behaviors in response to negative affect should be closely monitored, given the association with long-term maintenance of eating pathology.

## Figures and Tables

**TABLE 1:** Demographic information.

Variable	n (%)
Ethnicity/Race	
White	57 (83.8)
Hispanic or Latino	4 (5.9)
Black	5 (7.4)
Native American or American Indian	0 (0.0)
Asian/Pacific Islander	2 (2.9)
Other	
Education at Follow-Up (*n* = 48)	
High school graduate/GED	1 (2.1)
Some college	4 (8.3)
Associate’s degree	1 (2.1)
Bachelor’s degree	19 (39.6)
Some graduate education	12 (25.0)
Master’s degree or higher	11 (22.9)
Marital Status at Follow-Up (*n* = 48)	
Single/never married	24 (50.0)
Married/living with partner	21 (43.8)
Divorced	3 (6.3)

**TABLE 2:** Mean and standard deviation of global eating pathology and negative urgency at baseline and follow-up.

Pooled estimated mean (SD)				
	Baseline global eating pathology	Follow-up global eating pathology	Baseline negative urgency	Follow-up negative urgency
Bulimia nervosa (*n* = 36)	3.97 (0.87)	1.76 (1.46)	2.80 (0.54)	2.33 (0.78)
Purging disorder (*n* = 32)	3.38 (0.87)	2.29 (1.41)	2.59 (0.59)	2.58 (0.63)
Total Sample (*N* = 68)	3.69 (0.92)	2.01 (1.22)	2.70 (0.57)	2.45 (0.64)

## Data Availability

The data that support the findings of this study are available from the corresponding author upon reasonable request.
